# A research framework for the United Nations Decade of Healthy Ageing (2021–2030)

**DOI:** 10.1007/s10433-021-00679-7

**Published:** 2022-01-12

**Authors:** N. Keating

**Affiliations:** 1 Global Social Issues on Ageing (GSIA), International Association of Gerontology and Geriatrics, Buenos Aires, Argentina; 2 Centre for Innovative Ageing, Swansea University, Swansea, UK; 3 Research on Ageing, Policies and Practice (RAPP), University of Alberta, Edmonton, Canada; 4 Optentia Research Unit, North-West University, Potchefstroom, South Africa

**Keywords:** UN Decade of Healthy Ageing, Research framework, Environments, Life courses, Wellbeing

## Abstract

The mission of UN Decade of Healthy Ageing (2020–2030) is to improve the lives of older people, their families and their communities. In this paper, we create a conceptual framework and research agenda for researchers to knowledge to address the Decade action items. The framework builds on the main components of healthy ageing: Environments (highlighting society and community) across life courses (of work and family) toward wellbeing (of individuals, family members and communities). Knowledge gaps are identified within each area as priority research actions. Within societal environments, interrogating beliefs about ageism and about familism are proposed as a way to illustrate how macro approaches to older people influence their experiences. We need to interrogate the extent to which communities are good places to grow old; and whether they have sufficient resources to be supportive to older residents. Further articulation of trajectories and turning points across the full span of work and of family life courses is proposed to better understand their diversities and the extent to which they lead to adequate financial and social resources in later life. Components of wellbeing are proposed to monitor improvement in the lives of older people, their families and communities. Researcher priorities can be informed by regional and national strategies reflecting Decade actions.

## Introduction

In recent years, we have witnessed the growth of international debates about paradigms to frame action on population ageing. The debates have been populated with conversations about whether increasing proportions of older people comprise a demographic dividend (Fried and Rowe 2020); and how major global shocks such as pandemics, mass migration and climate change affect states, their economics and their people (Carmody et al. 2021). They have raised fundamental questions of citizenship, exclusion and inequalities (Hallegatte and Rozenberg 2017).

United Nations (UN) agencies have been influential in shaping these global discourses. They have established a values agenda, with principles for people and their environments that set a moral compass for ageing. They have created a framework for healthy ageing that positions older people within these environments and articulates processes and outcomes.

The UN Decade of Healthy Ageing (2021–2030) brings together these agendas toward a better future for older people. Its plan of action signals shared accountability for “concerted, catalytic and collaborative action to improve the lives of older people, their families and the communities in which they live” (WHO 2020). Academics are held responsible along with governments, civil society, international agencies, professionals, the media and the private sector.

The purpose of this paper is to propose a roadmap for researchers across world regions to address this global challenge. The roadmap is framed specifically around the values, actions and outcomes embedded in the Decade Report and Action Plan and the knowledge that is needed to support them. It has a social science lens, informed by the main components of healthy ageing: environments across life courses toward wellbeing.

The paper is structured in four sections.

- Principles underlying the Decade of Healthy Ageing. Synopses are presented of the beliefs and values inherent in related UN documents that establish the moral compass for the Decade and its sense of urgency for action; and the ideas and approaches that form the conceptual compass to enhance healthy ageing.
- Priority areas for action described in the Decade of Healthy Ageing Plan of Action (WHO 2020). The four action areas have been endorsed by WHO member states and provide the broad guidelines against which countries can measure their progress.
- Framework and knowledge gaps. We articulate the conceptual approaches underpinning the Decade and priority areas for research to inform the Decade agenda.
- Toward 2030. In the final section of the paper, we bring together these components, discussing how the research agenda can contribute to the mission of “improving the lives of older people, their families and the communities in which they live”.

## Principles underlying the UN Decade of Healthy Ageing

For 75 years, the United Nations (UN) and its specialist agencies including the World Health Organization (WHO) have had as their mission fostering of global cooperation around economic, social and humanitarian challenges and human rights and freedoms. The Charter of the United Nations (UN no date) established its core purpose to maintain peace and security. It also set the foundations for a broad values agenda that includes universal respect for human rights, wellbeing and fundamental freedoms for all.

The UN Sustainable Development Goals (2015) reflect this history. With their mission to leave no one behind, they create a vision for inclusion of all people across all world regions. The SDGs focus on people and on environments, with broad aspirations to end poverty, protect the planet and improve the lives and prospects of people in all world regions. They were adopted by all UN Member States in 2015, forming part of the 2030 Agenda for Sustainable Development.

The values stance reflected in the SDG document provides a moral compass for moving forward. Presented as a universal agenda, the dignity of individuals and inclusion of all nations and people are emphasized. Core principles include human rights for all, gender equality, social justice and equitable access to resources. Both people and environments are central, although the interconnections among them are implicit. There are no goals specifically related to older persons. However, ensuring healthy lives and promoting wellbeing (SDG3) foreshadows healthy ageing initiatives. Older persons are mentioned in the context of reaching those who are furthest behind. The language used is that of vulnerable groups — older persons along with children, youth, persons with disabilities, people living with HIV, indigenous peoples, refugees, internally displaced persons and migrants (United Nations 2021).

The World Report on Ageing and Health (WHO 2015) focuses on ageing and older people in the global agenda to leave no one behind. Following the SDGs and initiatives focused specifically on older persons such as the International Year of Older Persons (1999), its approach also is rights-based although its language marks a shift from older people as vulnerable, to older people as excluded. The change is important, moving the discourse toward the ways in which environments often impede, but indeed could foster healthy ageing. Terms such as ageism and inequity are used throughout, illustrating this perspective and foreshadowing action items in the Decade of Healthy Ageing. A key contribution is its’ focus on healthy ageing, providing a conceptual compass which is based on environments, in life courses and in wellbeing.

Environments are presented as an approach to understanding healthy ageing as a process of creating a good fit between individuals’ intrinsic capacity (especially physical and mental health) and their environments (home, community and society). A core assumption is that those with chronic conditions can manage well if their environments are sufficiently supportive. It foreshadows actions calling for global changes to environments to foster inclusion and reduce inequalities.

The life course approach is invoked to underscore inequalities among older people; to argue that these arise from the cumulative impact of advantage and disadvantage across people’s lives; and to label as inequities those differences that are unjust and must be addressed. The use of the term ageing reflects the assumption that lifelong processes shape late-life experiences.

Wellbeing is the third element of the approach to healthy ageing. Wellbeing is described as “the total universe of human life domains…that make up what can be called a “good life” (WHO 2015, p 244). A core assumption is that determination of a good life is grounded in the person’s assessment of their ability to be and do what they most value. By virtue of it being positioned as an outcome of healthy ageing, wellbeing becomes central to determining the success of the Decade of Healthy Ageing actions.

## Priority areas for action

The UN Decade of Healthy Ageing brings together these agendas with a call to action. Its goal is to increase the significance of ageing, create urgency to act and generate change that transforms population ageing from a challenge to an opportunity (WHO 2020). The argument is compelling. The projected rapid rise in numbers of people age 60 and older from 1 billion in 2019 to 2.1 billion in 2050 underscores the significance of population ageing. Urgency is underscored by the fact that the majority of older people are in low- and middle-income countries. Many older persons have no access to the resources necessary for lives of meaning and dignity and confront multiple barriers that prevent their full participation in society. Core principles in the document are consistent with human rights and social justice themes.

The priority areas for action that form the basis of the report were established through extensive consultations with WHO member states, UN agencies and non-state actors. The action agenda is based on broad consensus of their importance and commitment to their implementation. The report highlights four key areas for action, each with directives to create policy, develop programs and build evidence. The Decade of Healthy Ageing was endorsed by the United Nations in December 2020.

Action 1. “Changing how people think, feel and act toward age and ageing” (WHO 2020, p 9). This action is described as challenging negative attitudes about age which are common in all societies. The argument is that reducing ageism is important given its pervasive impact on the way problems are seen, priorities are assessed, and solutions are determined. Ageism marginalizes older people, reducing appreciation of their social capital and limiting their access to services. There is a call for research on the social and economic implications of ageism and on the contributions of older people.

Action 2. “Ensure that communities foster the abilities of older people” (WHO 2020, p 9). This action is described as fostering physical, social and economic environments that are good places to “grow, live, work, play and age” (WHO 2020, p.9). The argument here is that age-friendly communities include older people; enable them to age where they want to be; and foster autonomy, dignity and wellbeing of older people and their family carers. There is acknowledgement that these actions must be undertaken with available human, material and financial resources. There are calls for building knowledge and understanding of age and ageing, developing tools to monitor progress in developing age-friendly environments and collecting geographically disaggregated data.

Action 3. “Deliver person-centered, integrated care and primary health services responsive to older people” (WHO 2020, p 12). The rationale for this action is that older people need access to essential health services that are non-discriminatory and do not create financial hardship. The goal is universal health coverage. A competent workforce, appropriate legislation and sustainable funding are essential. There are calls for research to develop measurement tools for healthy ageing, on evidence gaps in clinical data, and on changes in health systems.

Action 4. “Provide access to long-term care for older people who need it” (WHO 2020, p 14). The argument in support of this action area is that declines in intrinsic capacity can limit people’s ability to care for themselves and participate in society. Access to long term care (LTC) helps people maintain their functional ability and live in dignity. The goal is that every country should have a system of long-term care that helps people maintain relationships and take part in activities that are meaningful for them. Reliance on families to provide LTC is seen as unsustainable and as inequitable notably for women. There are calls for comparative research on best LTC funding models in various resource settings and contexts, on cost effective interventions, and on the impact of long-term care on the wellbeing of recipients.

## Framing the research agenda

The UN endorsement of the Decade places a global spotlight on ageing. It that provides a timely opportunity for researchers to articulate and support its mission by fostering theoretical conversations and empirical advancements that will encourage coordinated, global research efforts to understand the contexts, processes and outcomes of healthy ageing. The research agenda we propose is based on the core concepts of environments, life courses and wellbeing which reflect foundational principles of the approach to healthy ageing. It is grounded in the values and action items in the Decade agenda.

### Environments

“Environments play a crucial role in promoting healthy ageing and ensuring no older person is left behind…Moreover, all domains of environments need to be assessed including support and relationships, attitudes, broader services, systems and policies” (WHO 2021, p 67).

From the SDGs through the Decade of Healthy Ageing and its action plan, environments have been presented as important in people’s lives. The emphasis on environments in the World Report on Ageing and Health (WHO 2015) reflects a view that the contexts of ageing can influence wellbeing and exacerbate or reduce late-life inequalities. This perspective is grounded in human ecological assumptions that people have various capacities to act upon or adapt their environments and that person-environment fit occurs when environmental demands and opportunities are balanced with individual capabilities and resources (Bigonnesse and Chaudhury 2020). Two further assumptions illustrate its’ values base: that responsibility for supporting those with limited agency must be shared; and that a goal is to identify and create evidence of those who are rendered invisible by contexts that exclude (Keating et al. 2021a, b).

Environments are social constructions, conceptualized as contexts or settings that are relevant to understanding a phenomenon of interest (Stephens et al. 2019). By naming specific environments, researchers declare research priorities and the scope of knowledge and knowledge gaps to be addressed within them. In this first part of the proposed research framework, we present two environments that are central to the phenomenon of healthy ageing: Society and Community. They are informed by the mission of the Decade and its main action areas. They reflect the assumption in social ecological models that environments are more or less distant from individuals (Keating and Phillips 2008). They are parsimonious, chosen to reflect key components of the values, beliefs, settings and relationships embedded in the Decade Action Plan and that shape experiences of ageing (Gelso 2006).

#### Societal environment

Societal environments are macro contexts that comprise beliefs and values of societies. It is within the societal environment that we find “values about age and ageing, about who is seen to be deserving of full citizenship and about how these cultural ways of knowing frame courses of action with a society” (Keating and Cheshire-Allen 2021). These value systems are important in influencing the goals set by governments, their policy actions and the ways in which individuals are affected and respond to them (Muers 2018). Two belief systems reflected in Decade Actions are ageism (Action 1) and familism (Action 4).

Ageism is “the complex, often negative construction of old age” (Ayalon and Tesch- Römer 2018, p 405) and a core element of the Decade action agenda. At the societal or structural level, ageism is the explicit or implicit bias against older persons that is embedded in societal norms and values and expressed in the policies, practices and actions of social institutions (Chang et al. 2020; Makore and Al-Maiyah 2021). The call for a campaign to end ageism is an indication of beliefs that ageism truncates the ability of ageing individuals to be fully included in their societies and is a hidden source of inequity (Kilaru and Gee 2020). Research and conceptual development on social exclusion has begun to reveal the ways in which societal beliefs and actions can exclude older people from full citizenship in areas such as services, amenities, mobility, social relations, material and financial resources and civic participation (Walsh et al. 2017).

The research backdrop to reducing societal ageism lies in understanding its development, persistence and diversity across societies (Marques et al. 2020). We have relatively good knowledge of these domains for individual ageism (see for example, WHO Global Report on Ageism 2021). Less is known about these at the structural level—an important knowledge base if we are to understand the likelihood that governments might pass human rights legislation that addresses age discrimination or modify existing instruments that permit it (WHO 2021 January).

Two areas that would benefit from further conceptual development and empirical examination at societal/institutional levels are positive ageism and implicit ageism. Positive ageism positions older people as worthy but needing nurturing and protection, making them subordinate to others who know best (Vervaecke and Meisner 2021). The negative impact of such beliefs was evident during the early phases of the Covid-19 pandemic. Many countries placed greater restrictions on older persons, accompanied by public communication that was patronizing and homogenizing, depicting all older adults as ‘vulnerable’ members of society (Fraser et al. 2020).

Implicit ageism is more difficult to identify and thus challenge. It includes what might be called averted ageism, arising from society’s gaze being on what are seen as more pressing issues such as the enablement of youth (Roux and Viljeon 2018). As scarcity of resources increases, especially in the face of increased numbers of older people, tensions over resource allocation lead to higher rates of ageism (Goldani 2010; Marques et al. 2020). Further, articulating societal approaches to ageism will provide a solid basis for tracking progress toward reducing its impact across countries and settings.

We include familism as the second set of societal beliefs and values comprising the main constructs in the societal environment. Familism is not named specifically in the Decade Action Plan, though its principles are alluded to in such statements as how holding families responsible for care of their older members is both unsustainable and inequitable. The pervasiveness of familism and its’ often-implicit assumptions make it both important and challenging to deconstruct. Like ageism, familism shapes lives.

Familism is beliefs about the centrality and responsibility of families for their individual members (Mucchi-Faina et al. 2010). At the societal level, it is reflected in the extent to which the state views families as responsible for the welfare of their members. Family responsibility may be entrenched through the absence of public policy support (familism by default); or active enforcement via policies such as family responsibility legislation, income transfers or workplace leaves (supported familism) (Ting and Woo 2009; Saraceno 2016). In periods of economic recession, there is a retreat to familism among welfare states (León and Pavolini 2014).

Outside of the welfare states in the global north, familism is often expressed as a core societal belief. In Latin America, familism has been described as an important cultural construct that prioritizes personal obligation to family needs (Hernandez and Bamaca-Colbert 2016). Button et al. (2018) speak of a widespread belief in kinship obligations in post-apartheid South Africa, with large numbers of people dependent on kin for either financial support or personal care. In turn, the so-called Black Tax (Webb 2021) illustrates the expected reciprocal support from younger people to family members.

The idea that families should be solely responsible for the care of their members has been described as “simultaneously commonsensical and strange” (Poletta 2018, p 230). Challenges to ideas of familism as benevolent have come primarily from researchers studying family care. There is considerable evidence that taken for granted assumptions about family care work as “natural and preferred” (Kemp 2021, p 145–146) come at high cost to carers’ labour force engagement (Casado- Marín et al. 2011), physical and mental health (Bauer and Sousa-Poza 2015) and family relationships (Broese van Groenou and De Boer 2016; Keating and Eales 2017). There has been less examination of the impact of familism on older adults or on families in the global south.

Levels of familism and beliefs about their positive impact on older persons and their families warrant further consideration. The commonsensical aspect of familism seems well-engrained, though we believe it has been insufficiently examined across geographic settings and policy contexts. Lack of action to address familism may be akin to familism by default (Kodate and Tinomen 2017), in which families are presumed to be in the best position to care for older members regardless of their ability, availability or suitability—a belief that it challenged in the Decade action on long term care.

#### Community Environment

Communities are most often described as meso contexts of physical and social settings in which people live their lives (Wahl and Oswald 2016). They include built and natural features, services and infrastructure and points of connection among residents (Andrews et al. 2013) and are viewed as having potential to provide a sense of place and of belonging (Obadia 2015; Wiles et al. 2017). Communities are a central part of the discussion of environments of healthy ageing (Action 2). In the language of the Decade Action Plan, there is an expectation that communities will be environments that are good places to “grow, live, work, play and age” (WHO 2020 p 9).

Environmental gerontologists have been instrumental in providing evidence of the positive role of built environments and belongingness in the wellbeing of older persons (Ahn et al. 2020; Moyano-Díaz et al. 2021). Yet research evidence by no means presents communities as universally good places to grow old. Ageing in place can embed disadvantage for people in settings with poor infrastructure, precarious housing, fear and mistrust (Finlay et al. 2020; Bates et al. 2019). Researchers and theorists seeking to understand social exclusion of older persons have articulated domains and processes through which older people may be denied full participation in their communities, regardless of level of community resources (Walsh et al. 2021).

A research issue that emerges from these findings is the extent to which communities differ in their ability to be supportive to a diversity of older residents. Community inequalities are particularly relevant to the Decade action item on community supportiveness given the growth of age-friendly approaches that establish domains within which communities are encouraged to act to meet the needs of their ageing populations (Sis et al 2020). Yet there is evidence that people with the greatest need for age-friendly interventions do not always reside in places with the capacity to implement them (Golant 2014). Researchers have articulated a need to better understand the ability of community structures and systems to address age-friendly principles (Rémillard-Boilard et al 2021; Winterton 2016).

A next step in understanding communities as contexts for healthy ageing is to investigate the extent to which communities themselves may be characterized by vulnerability (Skinner et al. 2016). Researchers have argued, for example, that rural communities may be poorly equipped to address older peoples’ needs given population decline, remoteness, limited fiscal resources and reliance on volunteers (McCrillis et al. 2021). Placing communities within macro contexts such as pandemics, climate change and political instability will also add considerably to our understanding of drivers of community capacity.

### Life courses

“We frame ageing within the life course” (Beard 2018, p A1).

In his reflection on the work of the WHO in formulating its approach to ageing, Beard underscored its’ emphasis on events and transitions that shape later life. The term Healthy Ageing was chosen to reflect the belief that if more people are to experience a good old age, we must incorporate processes across time as lives unfold. Researchers agree that if we want to fully understand later life, we must situate it within the socially constructed nature of the life course (Holman and Walker 2020).

A core assumption of life-course theory is that life pathways create the structure and rhythm of individual lives (Dannefer and Kelley-Moore 2009). Life course theorists have long been engaged in specifying these pathways, arguing that there is no singular life course, but several domains, each with transitions that punctuate it (Elder 1985; Elder and George 2016). In this second component of the research framework for a Decade of Healthy Ageing, we present life courses that we believe are especially relevant to understanding healthy ageing.

The Decade Report and Action Plan is grounded in pathways of intrinsic capacity. In its report on life courses of health, the Pan American Health Organization (2021) describes pathways of physical and mental health that diverge across the life course, leading to differences in risk of disease and healthy life expectancy. While the overall shape of health trajectories is hypothesized, turning points or transitions are not articulated. Nonetheless, the authors argue that the shift from health as a status to health as a process broadens the epidemiological model of health through its recognition of “biological, psychological, physical, social, and environmental influences that operate from conception to death” (PAHO 2021, p 23). Further specification of health trajectories is important in informing the Decade Action item of delivering essential health services (Action 3).

Conceptualizing health as a life course process, creates an opportunity to explicitly incorporate other life course domains. As with environments, we have yet to fully explore which life courses are most critical to healthy ageing. While the possibility for differentiated life course trajectories to influence later life outcomes is clear, the extent to which this happens as people’s lives unfold has not been sufficiently conceptualized (Walsh et al. 2020, p 2324). Further articulation and exploration of these pathways will illustrate cumulative advantage or disadvantage that are the precursors to late life inequalities.

We propose two additional life course domains to frame ageing within the life course: Work and Family. Each is important in shaping the lives of adults as they age. Each has transitions and trajectories that underscore the relevance of changes across time and their contributions to diversity in later life outcomes (Alwin 2013). In keeping with the focus in the Decade Action Plan, gaps in knowledge of transitions in the second half of life are emphasized.

#### Work life courses

Work trajectories have been conceptualized as a series of labor market states whose components include transitions between jobs, movement in and out of jobs and the overall timing of these shifts within individuals’ careers (Liukkonen et al. 2009; McDonough et al. 2017). There is a widely held belief that employment life courses have become more complex, more unstable and less predictable (Van Winkle and Fasang 2017), although this assumption has yet to be tested across the entire employment life course.

There has been recent interest in later life labor force engagement and exit that has illustrated difficulties for older workers in transitions such as re-entry into the labor force after a period of absence (Visser et al. 2018); and the extent to which types of retirement have differential impact on wellbeing (Radó and Boissonneault 2020). Other research has provided insights into transitions and processes across other elements of the employment life course. Scholars have articulated the start and end points of employment pathways through examination of how national policies and programmes structure age of (first) entry into and (final) exit from the labour force (Larsson and Stattin 2015; Clark et al. 2017). Diversity among work pathways has been explored through micro-transitions between employment to unemployment (Hägglund and Bächmann 2017), full time and part time work (Van Winkle and Fasang 2017) and sequential precarious employment (Raymo et al. 2011). Analyses across life courses of work with specific characteristics such as being precarious (Ní Léime and Street 2019) or physically demanding (Nicholas et al. 2020) have illustrated how work characteristics can have a cumulative effect on later life outcomes of poverty and illness/disability.

The next step is to map transitions and trajectories across the full span of a work career. Ní Léime and Street (2019) argue that there are two components needing further development. The first is the span of a working life. Research under the rubric of extending working lives (Lain et al. 2020) has illustrated advantages and disadvantages of lengthening the later part of the employment life course. There has been little research on how late-career extensions influence the overall length of working lives. One illustrative report raises a cautionary note about assuming benefits arise from long labor force careers. The authors found that across countries in Europe, those with the lowest expected years of labor market engagement have the highest life expectancy (Hytti and Valaste 2009) suggesting that shorter working lives are afforded to those who are most privileged.

The second component is the demands made on that working life. Features of work life courses may comprise a set of conditions that render work lives particularly challenging. How does long term engagement in precarious work with little security or benefits and low pay, result in lives infused with uncertainty and instability (Campbell and Price 2016)? In what ways does lifetime occupational engagement in physically and cognitively demanding jobs limit work capacity later in life (Nicholas et al. 2020)? Which workers are most advantaged by job flexibility and at what points in their employment life course (Kossek and Lautsch 2018)? These examples illustrate evidence gaps in how these and other demands shape how life courses of work will unfold.

There is much scope for further conceptual and empirical work to address knowledge gaps in life courses of work. We highlight one that we believe is relevant to the Decade of Healthy Ageing principle of reducing inequalities. It is to adopt definitions of work that incorporate the majority of workers in the global population who work in the informal sector (Bonnet et al. 2019; Mitra 2015). Informality encompasses a wide range of jobs and economic activities without work-based social protection where workers are usually poorer and more vulnerable than workers in formal employment (Bonnet et al. 2019). Importantly, such definitions could provide a context for examining life courses that might include precarity in both formal and informal employment (Burchielli et al. 2014; Siegmann and Schiphorst 2016). Such examinations could provide the basis for understanding how over time, precarious work might be associated with precarity as a general condition of later life (Campbell and Price 2016).

#### Family life courses

Life course perspectives on families have long been a central feature of family scholarship. Structural approaches featuring changes in family composition are foundational to much of this theorizing. An early example posited a succession of stages through which families pass during their life span. Marriage marked family formation; children were its main transitions; death of a spouse the final exit (Glick 1977; Rogers 1964). This so-called standard American family (SNAF) was challenged as an ideological code that reproduced itself in beliefs about ideal and deficit families (Marotz-Baden et al. 1979; Smith 1993). While family life course scholarship has developed considerably since this early thinking, it is worth noting that a major theme in the current concern about wellbeing of older people is the availability of children to provide support.

Contemporary family life course scholarship also draws on structural transitions, but with an assumption of diversity of family forms. Scholars have shown increased variation in family transitions resulting from policy changes such as the liberalization of laws regulating marriage and divorce (Jowett 2017) and changes in social mores such as those related to cohabitation (Stoilova et al. 2017) and fertility (Engin et al. 2020). For the most part, each transition has been studied separately, providing data on the likelihood of being childless (Kreyenfeld and Konietzka 2017), ever-married (Keenan et al 2017), divorced (Couch et al 2020) or widowed (Schmitz 2021) during their lives. We know less about how patterns of these family states and transitions combine to create trajectories that may differ in their potential for late-life family connections.

Researchers have begun to address this knowledge gap, illustrating the usefulness of trajectories in documenting the extent to which families have become more diverse, uncertain or fragile. For example, in a study of patterns of “relationship turnover” among people age 15–45 in Europe, Perelli-Harris and Lyons-Amos (2015) found eight distinct trajectories of cohabitation, marriage and union dissolution. A similar study of transitions in “family formation” including being single, cohabiting, married and presence of children found five dominant patterns among people age 15–50 (Van Winkle 2018). Together these studies contribute to an understanding of diversity in segments of family life courses.

Documenting family transitions in the second half of life is new conceptual territory. It requires us to identify and incorporate transitions in partnership status and in generational membership, determining which are critical to understanding how family lives evolve. It places us in a position to interrogate the key family transitions across the entire life course and test their validity across countries and regions. In combination, this conceptual and empirical work would allow for a more systematic approach to understanding diversity in family trajectories and how these may lead to different family experiences for older persons.

While structures set the boundaries around family membership, family members live in relationship (Carr and Utz 2020; Landes and Settersten 2019). Family lives are linked through their interdependence over time through the transfer of material resources (Gilligan et al. 2018), though production in family enterprises (Rondi et al. 2019) and through care for grandchildren and for older family members (Hoang et al. 2019).

These linkages can be sources of ongoing support, although here too we see evidence of heterogeneity. Diverse family life courses have produced forms of relatedness (Silverstein and Giarrusso 2010) that can lead to late life exclusion (Gilligan et al. 2018). There is evidence, for example, that across family life courses in which couples have divorced, older men are less likely than women to receive material support from children (Maes et al. 2020). Siblings estranged over tensions in parent care may be unprepared to support each other in later life (Jensen et al. 2020; Keating et al. 2019). Older adults without children or partners have lower levels of conflict in their family networks, perhaps because their absence allows for more elective involvement in their families and more choice of supportive relationships (Giardin et al. 2018; Widmer et al. 2018).

Family life courses provide researchers an opportunity to address the theme in the Decade Action Plan of the place of families in the lives of older persons. The family life course assumption of family diversity, raises the question of the extent to which different family trajectories lead to inclusion or exclusion from supportive family relationships in later life. A next step is to complete the work of developing typologies of family life courses and validate them across world regions. In concert with this mapping of life courses of families, more attention needs to be paid to how family connections and relationships also evolve and the extent to which lives that are linked enhance quality of life for people in those relationships (Bengtson and Allen 2009).

### Wellbeing

“The total universe of human life domains…that make up what can be called a “good life” (WHO 2015, p 244).

From the Sustainable Development Goals through actions to enhance healthy ageing in the Decade Action Plan, wellbeing has been central to the narrative of desired outcomes. In this final section of the research framework, we return to the challenge presented in the introduction to the paper — that if wellbeing of older people, their families and communities are the outcome of healthy ageing, we need articulate approaches to monitoring our progress.

There is a large body of research on the wellbeing of older adults. To a great extent it has been grounded in quality of life (Fernández-Ballesteros 2011; Tseng et al. 2018). The World Health Organization definition is widely used: “an individual’s perception of their position in life in the context of the culture and value systems in which they live, and in relation to their goals, expectations, standards and concerns” (The WHOQOL Group 1998). Others have argued that objective factors including health and financial resources as well as social relations should be included (González et al. 2021; Karimi and Brazier 2016).

Together these perspectives comprise ideas of ‘being well’ reflected in people’s evaluations of whether their situation allows them to live the life they most value (McGregor 2007); and of ‘doing well’ through having sufficient material and social resources (Sen 1999; Tronto 2017). They are consistent with the environmental gerontology perspective of ageing as a process of calibrating person-environment fit over time (Wahl and Oswald 2016). They can provide a platform to further articulate healthy ageing as people’s ability to function well in the contexts in which they live (Michel et al. 2021).

There are longstanding debates about the ideal conceptualization of wellbeing (Gillett-Swan and Sargeant 2015; Mikton C, personal communication). Engaging in these debates goes beyond the scope of this paper which is to inform build knowledge about wellbeing to inform the UN Decade. These are to determine which groups will be the focus and to articulate the “being well” and “doing well” domains for each.

Two groups have been central to our understanding of outcomes of healthy ageing: older persons and families. The priority for conceptualizing wellbeing of older persons is to understand and support processes of healthy ageing that enhance their ability to be and do what they most value. Families are featured as caregivers to older persons with chronic health problems whose wellbeing is important to sustainability of care.

Details of material, relational and subjective domains of older persons and family members have been the subject of recent research. Based on evidence of resources that underpin people’s ability to live well, health, income and housing seem reasonably placed as core components of their material wellbeing (Bates et al. 2019; Gildner et al. 2019; Ng et al. 2017). Core elements of relational wellbeing include family members and friends (Vos et al. 2020), although we have much to learn about variation in the extent to which these relationships are supportive.

Incorporating knowledge of diversity in areas such as ambiguous or exploitive family relations or decreased friend networks in the face of illness and death are examples. A systematic review of wellbeing of family caregivers found that material resources (including labour force participation and care-related out of pocket expenses); and relational resources (including with the cared-for person, other family members, friends and co-workers) are most important (Keating et al. 2021a, b). There is merit in further empirical examination of what are the core resources and relationships in their lives.

The scope of subjective wellbeing needs additional critical examination. Many measures are situation specific such as health-related quality of life for older persons (Velarde-Jurado and Avila-Figueroa 2002) and caregiver burden for family caregivers (van Exel et al. 2004). If the ability to be and do what you most value is the outcome of interest, then a broader view of subjective wellbeing in life goals warrants consideration.

Which other family members of older adults might be included in this wellbeing theorizing? Priority could be placed on marginalized families such as migrants who relocate to escape political or economic instability, leaving behind older relatives too ill to travel (C. Licopantis, personal communication). How do we calibrate wellbeing of such family members with impoverished material situations, severed relationships and who may be suffering from exposure to repeated incidents of moral distress (Molendijk 2018)? More generally, what are minimally acceptable levels of wellbeing across the groups of individuals described here? The social justice component of this theorizing requires us to be unequivocal about what resources are necessary and how they will be distributed (Nussbaum 2002).

Community wellbeing is a key element of the Decade mission. However, despite the Decade Action calling on communities to foster the abilities of older people, there has been little exploration of the capacity of communities to do this, Wellbeing could provide a way to evaluate the sufficiency of community material resources (e.g., infrastructure and local economy) and relationships (e.g., sense of coherence and participation) (DeVerteuil et al. 2020; Ryser et al. 2021) as well as community narratives (e.g., as flourishing or left behind) (Li and Zehr 2020; Wiseman and Brasher 2008). Mapping diversities across these wellbeing domains could help us better understand the extent to which older people with the greatest need for age-friendly interventions do not always reside in places with the capacity to implement them (Cueva et al 2021; Winterton 2016).

## Toward 2030

The research framework presented here provides a structure for researchers who wish to target their research efforts to inform the UN Decade of Healthy Ageing. It builds on the main components of healthy ageing: Environments (highlighting society and community) across life courses (of health, work and family) toward wellbeing (of individuals, family members and communities).

Across world regions, countries are developing responses to the Decade Action Plan. These documents express commitment to the plan and signal priority areas where knowledge creation might be particularly timely in informing policy decisions to address Decade goals. For example, in its National Strategic Plan on Ageing, Indonesia stresses the importance of all action items (The Ministry of Justice and Human Rights the Republic of Indonesia 2021). A recent analysis of Indonesia’s ageing policies suggests priority areas related to Action item 1, specifically how ageism excludes older persons from late life income security; and Action items 1 and 4, how familist perspectives place care of older people in the private sphere (Lestari et al. 2021). Chile also endorses Decade action items, presenting its national plan as an opportunity to create public policies from a social healthcare and rights perspective (Gobierno de Chile 2021). The focus of the first part of its Decade strategy responds to action items 3 and 4: improving health care and long-term care for older persons (Ministerio de Salud 2021).

Countries in Africa have taken a different approach to articulating priorities in their region to address the Decade Action Plan. A draft set of priorities written by academics in the region and circulated widely for feedback from civil society organizations, is now being considered for adoption by the AU (African Union). The main priorities are consistent with understanding connections between life courses and wellbeing. Its core argument is that strategic investments across the life course will enhance capacities and wellbeing in older age and benefit both older and younger people. Eliminating ageism and ensuring communities and families have adequate capacities and resources are priorities linked to Action items 1, 2 and 4 (J. Hoffman, personal communication).

Other national and regional strategies may also provide indications of areas in which countries are prepared to act. For example, Canada’s Dementia Strategy (Public Health Agency of Canada 2021) calls for evidence of successful interventions (relevant to Action item 4) and of contributions of build and social environments to wellbeing of persons with dementia (Action item 2). The European Commission and WHO Regional Office for Europe’s Age-friendly environments in Europe (no date) emphasizes the importance of local and regional authorities making strong commitment to supporting older residents (Action item 2). Age Platform Europe (no date) is a source of decade research priorities from the perspective of older people.

While the UN Decade has global research, evidence to support understanding of environments, life courses and wellbeing will be grounded within countries and regions. Taking global diversity and global voices seriously requires questioning assumptions around ideal life course pathways (Mokomane 2021); including how intersectionalities of gender, race and social class may contribute to widening inequalities across life courses (Folbre 2020); and changes in the fit between persons and their environments over time might comprise tipping points in later life precarity (Keating et al. 2013; Urbaniak and Walsh 2019). We must be mindful of the moral compass set out in the Sustainable Development Goals. It requires us to see inequalities and to foster inclusion. As researchers what we choose to study, the language we use and the data we collect will help determine the extent to which the lives of older persons are improved across the life domains articulated in this framework.
